# Nonvolatile Reconfigurable Synthetic Antiferromagnetic Devices Induced by Spin-Orbit Torque for Multifunctional In-Memory Computing

**DOI:** 10.3390/nano16070444

**Published:** 2026-04-07

**Authors:** Mingxu Song, Jiahao Liu, Zhihong Zhu

**Affiliations:** 1 College of Advanced Interdisciplinary Studies, National University of Defense Technology, Changsha 410073, China; songmingxu@nudt.edu.cn (M.S.); liujiahao20@nudt.edu.cn (J.L.); 2 Nanhu Laser Laboratory, National University of Defense Technology, Changsha 410073, China

**Keywords:** synthetic antiferromagnet, spin orbit torque, logic-in-memory, artificial neuron, artificial synapse

## Abstract

The proliferation of intelligent edge devices demands compact, low-power hardware capable of dynamically switching between sensing, logic, and learning tasks—a versatility that traditional multi-chip solutions fundamentally lack. Here, we demonstrate a reconfigurable spin–orbit torque (SOT) device based on an FeTb/Ru/Co synthetic antiferromagnetic (SAF) heterostructure. By modulating the input current amplitude, the device dynamically switches between two distinct operating modes: saturation and activation. In the saturation regime (>80 mA), deterministic magnetization reversal enables Boolean logic operations (AND, NOR). In the activation regime (<80 mA), gradual, non-volatile conductance modulation emulates synaptic plasticity. Benefiting from the strong antiferromagnetic coupling and near-zero net magnetization of the SAF structure, all operations are achieved without external magnetic fields. This single-device, dual-mode reconfigurable architecture establishes a new paradigm for high-density, low-power, multifunctional in-memory computing units, with promise for advancing adaptive edge computing chips.

## 1. Introduction

With the rapid advancement of artificial intelligence, the Internet of Things, and assisted driving, computing systems face the dual challenge of processing massive amounts of data while strictly controlling energy consumption [1,2]. In-memory computing fundamentally eliminates the overhead of data movement by enabling storage units to perform data processing simultaneously, positioning it as one of the most promising computing paradigms in the post-Moore’s Law era [3,4].

Among numerous memory computing candidate technologies, magnetic random-access memory (MRAM) based on spintronics has garnered significant attention due to its non-volatility, high-speed read/write capabilities, and compatibility with CMOS processes [5,6]. Specifically, spin–orbit torque (SOT)-based MRAM offers significant advantages over traditional spin–transfer torque (STT)-MRAM, including separated read/write paths, higher endurance, and faster switching speeds (sub-nanosecond level) [7,8,9]. The fundamental unit of SOT-MRAM typically consists of a heavy metal layer and a ferromagnetic layer [10]. A pure spin current is generated in the heavy metal layer via the spin Hall effect through the flow of the charge current, which then drives the reversal of magnetization in the adjacent ferromagnetic layer [11]. In recent years, utilizing SOT devices to achieve reconfigurable logic operations and neuromorphic computing has emerged as a research focus [12,13]. Fan et al. demonstrated the IrMn/Co/Ru/CoPt/CoO heterojunction by tuning both the in-plane exchange bias at the interface of IrMn and Co layers and the out-of-plane exchange bias at the interface of CoPt and CoO layers for applications in both multistate memory and programmable spin logic [14]. The researchers explore a ferromagnetic/antiferrimagnetic heterojunction as a basic device to perform multiple reconfigurable logic operations, while achieving high-density multi-state storage with 10 states. However, most of these studies are limited to implementing a single function (logic operations or synapse emulation) and often require external magnetic fields to break the symmetry of SOT switching, posing challenges to device integration and system stability [14,15,16].

Multifunctional integration within the same structure—achieving both digital logic operations and analog synaptic plasticity within the same physical unit—represents the ideal goal for realizing high-density, high-performance storage-computing chips [17]. Achieving this objective relies on the reconfigurability of the device’s operating region across different current magnitude ranges, meaning the device exhibits distinctly different physical behaviors at varying current levels. In the saturation region, high-current drive induces deterministic magnetic moment flipping, corresponding to binary logic states [18]. In the activation region, subthreshold currents achieve continuous conductance modulation through thermally assisted or domain wall motion, corresponding to analog synaptic weights [19,20]. SAF structures provide an ideal material platform for this purpose through strong antiferromagnetic coupling between two ferromagnetic layers, which can achieve near-zero net magnetization and enhanced thermal stability, enabling SOT switching without an external field [13,21,22].

Building upon the aforementioned foundation, this paper further explores reconfigurable memory computing functions within the operational regions of the same SAF devices. The key innovations include: (i) Dual-mode operation: within a single SAF device, seamless integration of digital logic operations (saturation region) and analog synaptic plasticity (activation region) is achieved by adjusting the current amplitude; (ii) Region-reconfigurable logic: Within the saturation region, multiple Boolean logic operations are constructed by leveraging the device’s deterministic flip characteristics, with dynamic switching between different logic functions achieved through input pulse combinations; (iii) Analog synaptic functionality: In the activation region, linear and non-volatile modulation of conductance is achieved through subthreshold multi-pulse accumulation effects, successfully simulating the long-term potentiation (LTP) and long-term depression (LTD) plasticity of a biological synapse. The dual-mode, zero-external-field reconfigurable architecture provides a novel device-level solution for developing high-density, low-power, adaptive in-memory computing chips suitable for edge computing.

## 2. Methods

Experiment sample fabrication and measurement:

A magnetic multilayer stack of Ta(2)/Ir(3)/FeTb(6)/Ru(1.1)/Co(1)/Pt(3)/Ta(2) (the numbers denote the thickness in nanometers) was fabricated on thermally oxidized silicon substrates using a magnetron sputtering system (AJA Orion 8). The base pressure of the main chamber was greater than 1 × 10^−8^ Torr and the Ar pressure was 3.0 mTorr. A 2 nm thick layer of Ta was used as an adhesive layer for preventing the oxidization of the underlying FeTb layer. The FeTb layer was synthetized through co-sputtering of the Fe and Tb targets. The multilayer film was patterned into a crossbar device with a channel width of 10 µm by using standard photolithography and ion beam etching. The magnetometry and electrical transport property measurements were performed utilizing VSM and a home-built electrical transport measurement system. The magnetization configuration images were taken by a polar MOKE imaging microscope from evico magnetics (Dresden, Germany).

## 3. Results and Discussion

**Figure 1:**
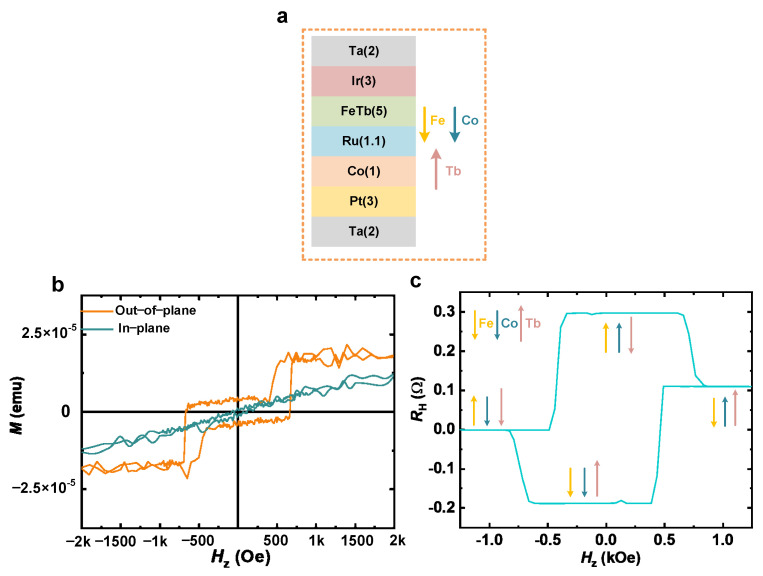
(**a**) Schematic illustration of SAF coupled through the interlayer-exchanged Ru layer. (**b**) Magnetization hysteresis loops under the out-of-plane and in-plane magnetic fields. (**c**) Anomalous Hall resistance *R*_H_ plotted as a function of the out-of-plane magnetic field *H*_z_ measured with a current of +1 mA.

Ta(2)/Ir(3)/Fe_0.61_Tb_0.39_(5)/Ru(1.1)/Co(1)/Pt(3) (thickness in nanometers) multilayers were deposited on the Si–SiO_2_ substrate utilizing a magnetron sputtering system, as schematically illustrated in Figure 1a, in which the compensated ferrimagnetic Fe_0.61_Tb_0.39_ film was made by co-sputtering Fe and Tb targets. The Ru layer mediates the interlayer exchange coupling in the form of an interlayer RKKY interaction between the Fe_0.61_Tb_0.39_ layer and the Co layer [23]. The 3 nm Ir layer in the multilayer stack serves multiple physical and structural roles. First, it acts as a capping/protection layer preventing oxidation of the underlying FeTb layer, which is prone to oxidation due to the high reactivity of Tb. Second, Ir is a 5d heavy metal with strong spin–orbit coupling, which can contribute to spin–orbit torque generation. Studies have shown that Ir possesses a spin Hall angle of ~0.005 and a spin diffusion length of ~1.2 nm, and its low resistivity helps reduce Joule heating compared to Pt-based devices. Third, the Ir/FeTb interface can enhance perpendicular magnetic anisotropy and Dzyaloshinskii–Moriya interaction, which may influence domain wall chirality and switching dynamics [24,25]. Although the present device relies primarily on uniform magnetization switching rather than domain wall motion, the Ir layer contributes to the overall spin transport and interfacial properties of the heterostructure. Quantitative current distribution analysis (Supporting Information, Table S1) reveals that ~70% of the total current flows through the Pt layer, which, due to its large spin Hall angle, generates the spin-polarized current that drives SOT switching. The Ir layer (~15% current) acts primarily as a capping layer with negligible spin current contribution, while the Co layer carries only ~5% of the current, confirming that the switching is not dominated by direct heating. Furthermore, both out-of-plane and in-plane hysteresis loops of the SAF sample were obtained by using a vibrating sample magnetometer (VSM) shown in Figure 1b, which confirms the presence of perpendicular magnetic anisotropy (PMA). The magnetic multilayer films were then patterned into a crossbar device using standard photolithography and Ar-ion milling to investigate the magnetoelectric transport properties. Square-shaped anomalous Hall resistance (AHE) loops as a function of the perpendicular magnetic field (*H*_z_) are shown in Figure 1c, which further confirms the presence of PMA. At the same time, the current-induced magnetization switching loops demonstrate four different arrangements of magnetic moments: (${\vec{M}}_{Fe-Tb}$↑ ${\vec{M}}_{Co}$, ${\vec{M}}_{Fe-Tb}$↑ ${\vec{M}}_{Co}$, ${\vec{M}}_{Fe-Tb}$ ${\vec{M}}_{Co}$, ${\vec{M}}_{Fe-Tb}$ ${\vec{M}}_{Co}$).

**Figure 2:**
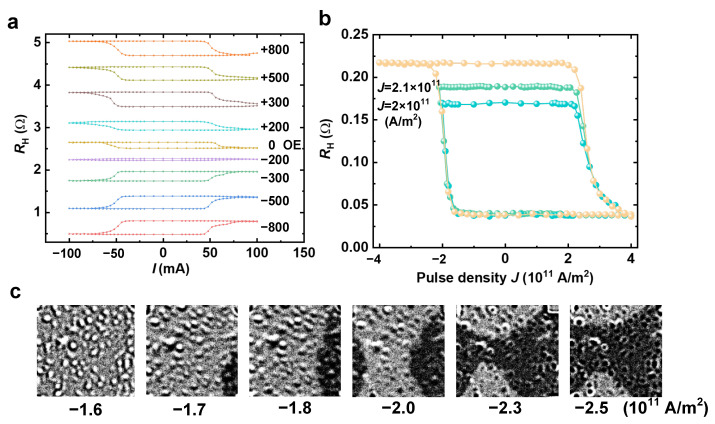
Field-free SOT switching and electrical/optical characterization. (**a**) Anomalous Hall resistance *R*_H_ as a function of injected current I under various auxiliary magnetic fields *H*_x_ (from −800 Oe to +800 Oe). The hysteresis loop at zero field (*H*_x_ = 0) exhibits clear switching with a resistance change ΔR ≈ 0.2 Ω, demonstrating deterministic field-free SOT switching. (**b**) *R*_H_ versus current density *J* measured with a fixed pulse width of 1 ms under zero magnetic field as the current pulse amplitude increases. (**c**) The p-MOKE images corresponding to magnetic domain nucleation and expansion.

To investigate the current-driven magnetization reversal behavior of the device, Anomalous Hall resistance *R*_H_ as a function of the injected current *I* was measured at room temperature. Figure 2a shows the *R*_H_ curves under different in-plane auxiliary magnetic fields *H*_x_ ranging from −800 Oe to +800 Oe. All curves exhibit distinct hysteresis loops, indicating deterministic magnetization reversal between positive and negative currents. Notably, even at zero auxiliary field (*H*_x_ = 0), the curve displays a pronounced loop with a resistance change ΔR ≈ 0.2 Ω between high and low resistance states, directly confirming the zero-field SOT reversal capability. This property originates from intrinsic symmetry breaking induced by strong interlayer coupling within the synthetic antiferromagnetic structure, along with potential exchange bias effects at the interfaces [26].

As the auxiliary magnetic field increases, the hysteresis loop gradually shifts, manifesting as a displacement of the reversal current direction. This shift aligns with the direction of the effective spin–orbit torque field. Shown in Figure 2b are the (major/minor) loops of the measured AHE resistances as a function of the pulse current density *J* with the duration being fixed at 1 ms. The current density was firstly swept from +4 × 10^11^ A/m^2^ to −4 × 10^11^ A/m^2^, and then reduced back to +4 × 10^11^ A/m^2^. In the following experiment, the current was swept from +4 × 10^11^ A/m^2^, and ended at a progressively smaller positive current. These curves clearly show multiple non-volatile AHE resistances during the progressive increase in current density. This impulse-regulated multi-state response provides the foundation for synaptic weight modulation in neuromorphic computing, where varying the amplitude or number of pulse currents can simulate conductance changes within the same device. A future practical implementation could benefit from integrating a magnetic tunnel junction (MTJ) as the readout element. The MTJ would provide a tunneling magnetoresistance (TMR) ratio exceeding 100%, substantially improving the readout margin compared to the present anomalous Hall effect scheme (~0.2 Ω), thereby reducing the sensitivity requirements of peripheral sense amplifiers. This work focuses on the functional demonstration of the SOT-SAF device, whereas integration with MTJs suggests a viable route toward high-density, low-power in-memory computing.

Figure 2c displays polar magneto-optical Kerr effect (p-MOKE) images that were obtained under different current pulse densities. Following the increased amplitudes of the current pulse, one can evidently identify that SOT switching takes place through domain-wall (DW) nucleation and expansion. Combining electrical and optical measurements, the deterministic SOT reversal of the device is achieved at zero field. Furthermore, by controlling the current density, multistate resistance can be realized, laying the foundation for subsequent logic operations and synapse emulation.

**Figure 3:**
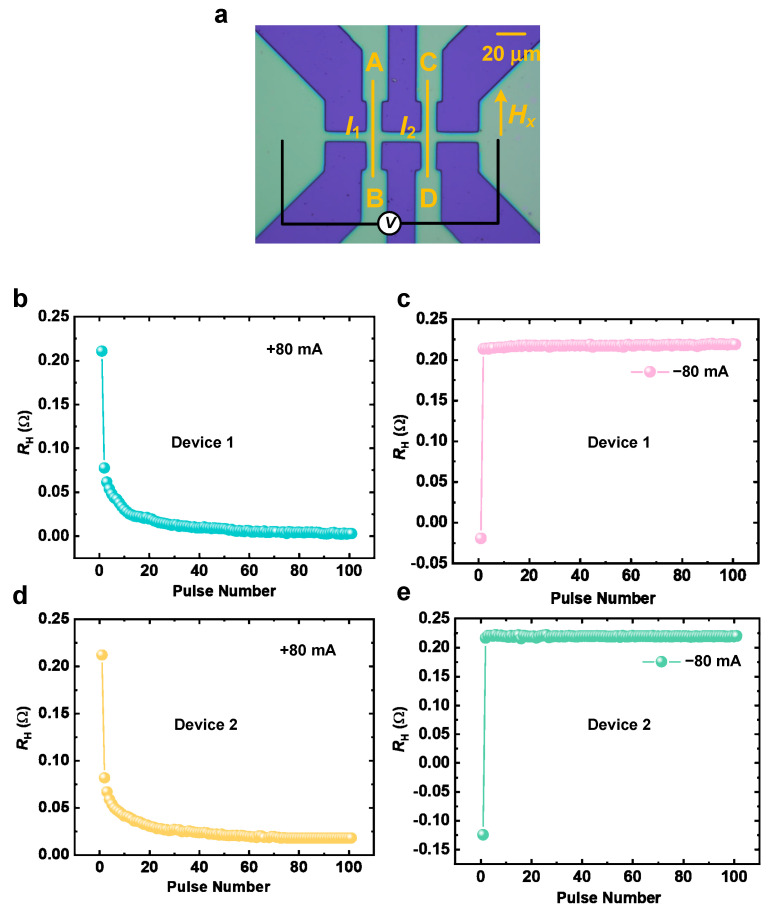
Deterministic switching stability and complementary behavior of the dual-element unit integrated by two Hall bars. (**a**) A schematic illustration of the spin logic gate design controlled based on the Si substrate by the path of the pulse currents. (**b**) Resistance evolution of element unit 1 under 100 consecutive positive current pulses (+80 mA). (**c**) Resistance evolution of element unit 1 under 100 consecutive negative current pulses (−80 mA). (**d**) Resistance evolution of element unit 2 under 100 consecutive positive current pulses (+80 mA). (**e**) Resistance evolution of element unit 2 under 100 consecutive negative current pulses (−80 mA). All pulse amplitudes are set to ±80 mA, which exceeds the critical switching current, ensuring complete and reproducible magnetization reversal. The opposite switching polarities of the two devices provide the foundation for reconfigurable logic operations.

In the aforementioned work, the SAF device was demonstrated to achieve deterministic SOT reversal at zero external field, with the reversal direction determined by current polarity and exhibiting asymmetry between positive and negative critical currents. This can be attributed to the device’s intrinsic exchange bias or interfacial asymmetry [21,27]. Building upon this foundation, the dual-element unit was integrated with two Hall bars, which are defined as elements 1 and 2, as shown in Figure 3a. *I*_1_ and *I*_2_ represent the currents passing through the corresponding elements. By controlling the current flow direction from B→A and D→C (positive), or from A→B and C→D (negative) with Hall voltage measured via lateral electrodes, the logic of the device can be programmed. When a pulse current exceeding the critical current is applied, *R*_H_ of each element can be rewritten according to the direction of the pulse current. Correspondingly, the total *R*_H_ of the dual-element device can be measured as the output signal. By setting a reasonable benchmark, it can complete Boolean logic functions using the dual-element device.

To validate reversal reliability and device consistency, we applied 100 consecutive saturated current pulses (±80 mA amplitude, exceeding the critical reversal current) to each device. Figure 3b,c show the response of element unit 1 under positive and negative pulses, respectively. After applying +80 mA pulses, element unit 1 rapidly enters a low-resistance state (LRS) and remains stable during subsequent positive pulses (Figure 3b). Likewise, after applying −80 mA pulses, element unit 1 rapidly enters a high-resistance state (HRS) and remains stable during subsequent negative pulses (Figure 3c). Figure 3d,e display the same response in element unit 2 under identical pulse conditions (Figure 3d,e).

In addition, the asymmetric switching dynamics observed in Figure 3 (gradual resistance change under +80 mA pulses versus immediate switching under −80 mA pulses) arise from the combined effect of the intrinsic bias field and the interfacial Dzyaloshinskii–Moriya interaction (DMI). The intrinsic bias field H_int_ originates from the antiferromagnetic interlayer coupling in the SAF structure. It breaks the degeneracy of the two switching directions, resulting in asymmetric critical currents: I_c+_ ~ −35 mA (LRS→HRS) and I_c−_ ~ +45 mA (HRS→LRS). This asymmetry sets the stage for the different dynamic responses [28]. The interfacial DMI at the Pt/Co and Ir/FeTb interfaces plays a crucial role in determining the switching mechanism under different current polarities [25,28,29]. In ferromagnet/heavy metal heterostructures, the DMI stabilizes chiral Néel domain walls and influences the effective magnetic fields during magnetization reversal. Recent studies have shown that anisotropic DMI can introduce pronounced asymmetry in the magnetization reversal process by breaking the symmetry of the switching trajectory. The interplay between the DMI and the intrinsic bias field determines the switching mechanism: When a negative current pulse (−80 mA) is applied, it drives the device from LRS to HRS. Although this direction has a smaller critical current (35 mA), the applied pulse amplitude (−80 mA) is significantly above the threshold. In this configuration, the spin–orbit torque (SOT) generated by the negative current is aligned with the intrinsic bias field (because the sign of the current determines the direction of the spin–orbit effective field). This alignment produces a strong, coherent torque that enables the magnetization to be reversed via coherent rotation, a fast process that can be completed within a single pulse. Consequently, the switching is immediate, as observed in Figure 3c,e. When a positive current pulse (+80 mA) is applied, it drives the device from HRS to LRS. This direction has a larger critical current (45 mA), and the applied pulse amplitude is only slightly above the threshold. More importantly, the SOT effective field generated by the positive current is opposed to the intrinsic bias field. This opposition forces the reversal to proceed via a domain-wall nucleation and propagation process, which is inherently slower and requires multiple pulses to complete, leading to the gradual resistance change observed in Figure 3b,d.

Additionally, the resistance change ΔR ≈ 0.2 Ω and the reversal polarity are highly consistent across both devices, demonstrating excellent repeatability and reliability in device fabrication. In summary, the experimental results in Figure 3 demonstrate that our fabricated SOT devices exhibit excellent consistency and reliability. By independently controlling current polarity, stable and reconfigurable state programming can be achieved without an external field, laying the foundation for developing high-density, low-power memory computing architectures.

By leveraging the complementary programming capabilities of the two devices, fundamental Boolean logic operations are further demonstrated. Before proceeding to the logic performance, it is necessary to define the logic values first. The absolute value of the pulse current is set to 80 mA. *I*_1_ and *I*_2_ are defined as Input 1 and Input 2, respectively. The input logic is defined by the polarity of the current pulses applied to each device: +80 mA represents logic “0” while −80 mA represents logic “1”. The output logic is determined by comparing the measured total resistance against a benchmark Hall resistance *R*th = 0.3 Ω. An output resistance above 0.3 Ω is defined as logic “1” while a resistance below 0.3 Ω is defined as logic “0”. This *R*th is based on typical values for single-device HRS (≈ 0.23 Ω) and LRS (≈0.02 Ω), ensuring all “0” state outputs fall below the threshold while “1” state outputs exceed it.

**Figure 4:**
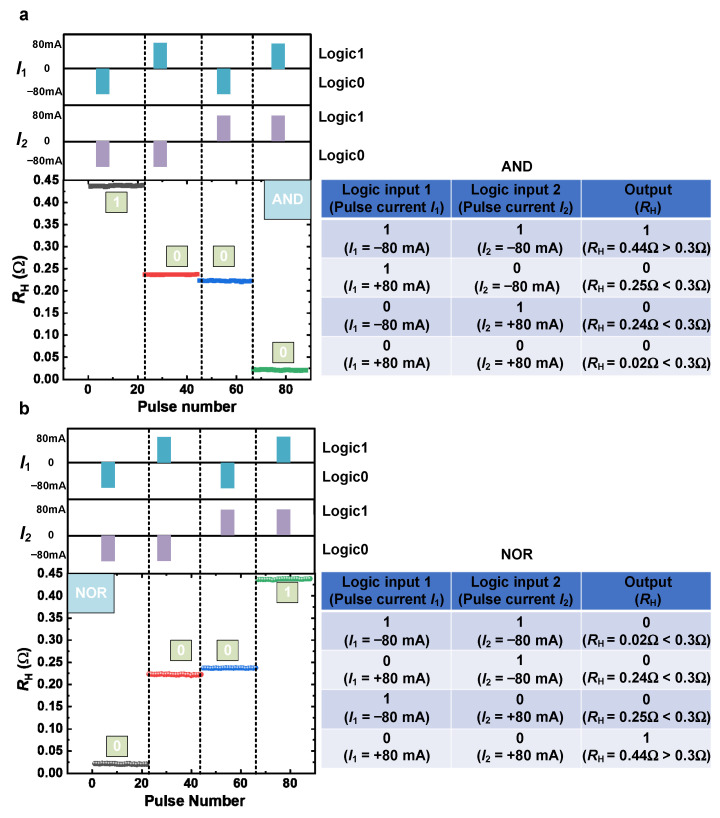
Demonstration of logic gate functions in the dual-element unit devices. (**a**) Timing diagram showing the output *R*_H_ as a function of pulse number and the corresponding truth table of the AND logic gate. (**b**) Timing diagram showing the output *R*_H_ as a function of pulse number for redefining the direction of current flow and the corresponding truth table of the NOR logic gate.

For the operation of the AND gate, the current flows in the direction of B→A and D→C (“→” represents the positive current direction). Figure 4a shows timing measurements for implementing AND gate operation to verify operational stability and repeatability. When *I*_1_ and *I*_2_ are set to −80 mA (Both Input 1 and Input 2 are logic “1”), elements 1 and 2 take on the HRS states and the total *R*_H_ of the dual element is measured to be 0.44 Ω (above the benchmark of 0.3 Ω). When *I*_1_ = +80 mA and *I*_2_ = −80 mA, element 1 is in the LRS state while element 2 is in the HRS state. The Hall resistance of the dual element is measured to be ≈ 0.25 Ω, which is lower than −0.3 Ω, and the logic is Output 0. Similarly, when *I*_1_ and *I*_2_ are set to +80 mA (both Input 1 and Input 2 are logic 0), elements 1 and 2 take on the LRS states. The Hall resistance of the dual element is measured to be 0.02 Ω, and the logic is Output 0 relative to the benchmark of 0.3 Ω based on the summarized truth table format. It can be observed that the SOT-based AND gate is realized by the pulse current without the participation of the magnetic field, and the output resistance remains highly stable throughout each phase without any degradation or drift, fully demonstrating the non-volatility and reliability of the logic operation.

As shown in Figure 4b, the NOR logic gate was also successfully implemented by redefining the direction of the current flow. In this case, the pulse current is set to flow in the direction of A→B and C→D. The core of this implementation lies in utilizing the inherent flipping characteristic of the devices, which can be programmed via current polarity. The consistent behavior of both devices allows us to arbitrarily combine their states (HRS or LRS) through simple pulse polarity selection, thereby generating different total resistance values. Beyond the AND/NOR gate example, the same hardware platform can be easily reconfigured into other logic gates (such as OR gates, NAND gates, etc.) by altering the combination of input polarities, offering new avenues for constructing large-scale, low-power in-memory computing architectures.

**Figure 5:**
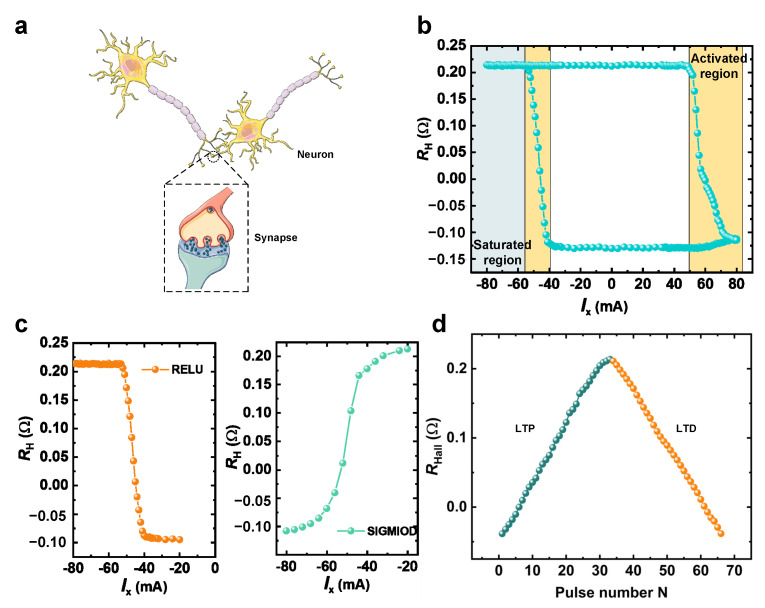
Dual-mode operation of the SOT device: from digital logic to neuromorphic computing. (**a**) Schematic illustration of a neuron and synapse implemented based on SOT device architecture. By operating the device in different current regimes, it can emulate either digital logic gates (saturation regime) or biological synaptic/neuronal functions (activation regime). (**b**) *R*_H_ as a function of injected current *I*, highlighting two distinct operating regions. The saturation regime (green shaded area) corresponds to current amplitudes above the critical switching current (|I| > *I*_C_) and the activation regime (yellow shaded area) corresponds to subthreshold current amplitudes (|I| < *I*_C_). (**c**) Neuron activation functions demonstrated in the activation regime. (**d**) Demonstration of LTP and LTD enabled by applying potentiation (depression) training pulse with a pulse width of 1 ms.

Beyond deterministic flip in the saturation region, the device exhibits tunable neuron and synapse emulation behavior in the subthreshold region. This “dual-mode operation” enables the SAF device to simultaneously support digital logic and analog neuromorphic computation, paving the way for multifunctional in-memory computing architectures. Figure 5a shows the schematic illustration of a neuron and synapse implemented based on SOT device architecture. When the device is biased in different current regions, it can function as a digital logic gate in “saturation mode” or simulate neuronal and synaptic functions in “activation mode”. Figure 5b displays the typical *R*_H_–I curves with the two operational regions highlighted in different colors. The green shaded area (saturation region) corresponds to the current amplitude range exceeding the critical switching current (*I*_C_ ≈ −40 mA). Within this region, the device reversibly switches between the HRS and LRS states under positive and negative current pulses, maintaining non-volatile state retention, which forms the basis for the logic operations demonstrated in Figure 4. The yellow shaded region (activation zone) corresponds to the subthreshold range where current amplitudes are below *I*_C_. Within this region, a single pulse is insufficient to fully reverse magnetization, but the cumulative effect of multiple pulses can progressively alter the magnetization state, enabling fine, analog-like resistance control. This control is non-volatile—the state persists after pulse removal and can be programmed by pulse number or amplitude.

Based on the characteristics of the activation region, two fundamental neuromorphic functions are further demonstrated. Figure 5c illustrates two typical neural activation functions. The “ReLU” (rectified linear unit) neuron in the left panel shows that when the input current exceeds a certain threshold, the *R*_H_ increases linearly with the input current. This behavior closely resembles the action potential generation in biological neurons when the membrane potential surpasses a threshold. The “Sigmoid” neuron in right figure depicts that *R*_H_ varies along an S-shaped curve with input current, which is one of the commonly used activation functions in artificial neural networks. Figure 5d illustrates the change in AHE resistance in both LTP and LTD versus the number of pulses. By applying 22 consecutive subthreshold negative/positive pulse sequences, the AHE resistance increases or decreases linearly with the number of pulses and the slopes for potentiation and depression are nearly identical. This continuous, non-volatile, linearly symmetric variation directly maps onto the synaptic weight update process in neural networks, forming the foundation for on-chip online learning. Notably, these two distinct functions (digital logic and analog neuromorphic computation) are realized on the same physical device, dynamically switched solely by varying the input current amplitude. When the current is applied in the saturation region, the device behaves as a binary digital switch, suitable for Boolean logic operations. When the current drops to the subthreshold activation region, the device functions as an analog adjustable element, suitable for neuromorphic computing. This “operational region reconfigurability” provides a novel device-level solution for developing high-density, low-power, multifunctional in-memory computing chips.

## 4. Conclusions and Outlook

This paper systematically investigates an FeTb/Ru/Co-based SAF device, demonstrating its multifunctional memory and computing applications under zero external magnetic field. Leveraging the dual-mode operation of the device, functional integration from digital logic to analog neuromorphic computation is achieved. In the saturation current region (|I| > *I*_C_), the device functions as a binary switch. By controlling the current polarity of the dual-element unit devices, AND/NOR logic operations are successfully implemented. In the subthreshold activation region (|I| < *I*_C_), the device exhibits continuous resistive modulation behavior, simulating ReLU and Sigmoid neural activation functions. It also enables linear and symmetric synaptic weight updates, providing a hardware foundation for on-chip learning. Notably, all these functions are implemented on the same device platform, dynamically switching operation modes solely by adjusting the input current amplitude. This fully demonstrates the device’s reconfigurability and potential for multifunctional integration.

## Data Availability

Data underlying the results presented in this paper are not publicly available at this time but may be obtained from the authors upon reasonable request.

## Supplementary Materials

The following supporting information can be downloaded at: https://www.mdpi.com/article/10.3390/nano16070444/s1, Figure S1: Size-dependent properties of SAF; Table S1: The calculated current distribution; Table S2: List of experimentally reported demonstrations of SOT-MTJ along with their key characteristics and properties.
