# Supplementary material for: Nonvolatile Reconfigurable Synthetic Antiferromagnetic Devices Induced by Spin-Orbit Torque for Multifunctional In-Memory Computing
Source: Nanomaterials (Basel). 2026 Apr 7;16(7):444. doi: 10.3390/nano16070444 (PMC13074596; doi:10.3390/nano16070444)
Supplement: Supplementary file 1 [file nanomaterials-16-00444-s001.zip › nanomaterials-4225399-supplementary.pdf]

Supplementary Information

Nonvolatile Reconfigurable Synthetic Antiferromagnets Devices Induced by Spin−Orbit Torque for Multifunctional In-Memory Computing

Mingxu Song [1,2], Jiahao Liu [1,2] and Zhihong Zhu [1,2,*]

[1] *College of Advanced Interdisciplinary Studies, National University of Defense Technology, Changsha 410073, China*
[2] *Nanhu Laser Laboratory, National University of Defense Technology, Changsha 410073, China*
***** Correspondence: authors. Emails: zhuzhihong@163.com;

S1. The quantitative estimation of the current distribution, layer resistivities, and the effective current density in the magnetic layers

1. Resistivity of Individual Layers

The resistivities of the individual layers were estimated based on literature values for sputter-deposited thin films and our own separate film measurements:

Layer	Thickness (nm)	Resistivity ρ (μΩ·cm)
Ta (bottom/top)	3/3	~ 180
Pt	3	~ 20
Co	1	~ 15
Ru	1.1	~ 12
FeTb	6	~ 150
Ir	2	~ 10

Noting that the direct literature values are limited for FeTb. We estimate ρ ≈ 150 μΩ·cm based on reports for amorphous FeTb alloys, which typically exhibit resistivities in the range of 100–200 μΩ·cm due to their disordered structure and strong spin-disorder scattering.

2. **Current Distribution in the Multilayer Stack**

Using a parallel conduction model (all layers conduct in parallel), the current fraction through each layer is:

$$f_i = \frac{t_i}{\rho_i} / \sum \frac{t_j}{\rho_j} \tag{1}$$

where t_i and ρ_i are the thickness and resistivity of layer i.

Layer	Thickness (nm)	Resistivity ρ (μΩ·cm)	Conductance (a.u.)	Current Fraction
Ta (bottom/top)	3/3	~ 180	0.017	~ 1 %
Pt	3	~ 20	0.150	~ 70 %
Co	1	~ 15	0.067	~ 5 %
Ru	1.1	~ 12	0.092	~ 7 %
FeTb	6	~ 150	0.133	~ 10 %
Ir	2	~ 10	0.200	~ 15 %

Table S1. The calculated current distribution.

From the current distribution analysis, it could be seen that the Pt layer carries approximately 70% of the total current. Combined with its large spin Hall angle, this confirms that the Pt layer is the primary source of the spin-polarized current that drives the SOT switching. In contrast, the Ir layer, although carrying about 15% of the current, has a very small spin Hall angle ($\theta_{SH} \approx 0.005$) and thus contributes negligibly to the spin current generation. Its main role in the stack is as a capping layer to protect the underlying FeTb from oxidation. The Co layer, which is the magnetic layer directly switched by the SOT, carries only about 5% of the total current, indicating that the switching is not dominated by direct current heating or other parasitic effects. These quantitative results provide strong support for the interpretation that the observed switching is indeed driven by SOT from the Pt layer, with well-separated roles for the other layers.

3. **Effective Current Density in the Co Layer**

For the critical switching current Ic ~ 40 mA and a Hall bar width of W = 10 μm, the total current density in the stack could be calculated to be $\boldsymbol{J}_{\mathrm{total}} \approx 1.8 \times 10^{11}$ A/m^2 by equation (1), where

t_{total} ~ 22 nm is the total thickness of the metallic stack. The effective current density in the Co layer (the layer that experiences the SOT) is 2×10^{11} A/m^2. This value is well within the typical range for SOT-driven switching in perpendicularly magnetized systems (10^{11} ~ 10^{12} A/m^2).

4. **Implications for SOT Mechanism Validation**

This quantitative analysis supports the interpretation that:

(1) The majority of the current flows through the Pt layer, which has a large spin Hall angle, generating a significant spin current.

(2) The effective current density in the Co layer is sufficiently high to induce SOT-driven switching.

(3) The observed switching behavior is consistent with typical SOT efficiencies reported in the literature .

S2. Size-dependent properties of SAF, associated energy consumption, and analysis of scalability

(1) **Energy estimation:** Using the critical switching current I_c ~ 40 mA, device resistance R ~ 100 Ω, and a pulse width of 1 ms (the condition used for the logic demonstration), the switching energy is $E = I_c^2Rt$ ~ 1.6×10^{-4} J. While this value is high due to the long pulse width used for proof-of-concept, the critical current scales linearly with device width (Figure S1). Reducing the width from 10 μm to 2 μm would lower I_c, and using shorter pulses (ns range) would reduce the energy by orders of magnitude, bringing it into the pJ range typical for SOT-MRAM.

(2) **Scaling discussion:** In addtion, most of the SOT-MRAM and SOT-based neuromorphic devices reported in the literature require an external in-plane magnetic field to achieve deterministic switching(Seen in Table S2). In such cases, the total energy consumption should include not only the electrical energy for the write current but also the energy needed to generate the auxiliary magnetic field. This field is typically produced by an on-chip current line or an external electromagnet, both of which consume additional power and add system-level complexity.

In our work, the field-free switching is intrinsically achieved through the antiferromagnetic coupling in the SAF structure. Therefore, no external magnetic field is needed, and the total system energy is solely determined by the current pulse used for writing. Even if we conservatively consider only the electrical write energy, our scaled device (1 μm width, 1 ns pulse) is estimated to consume about 64 fJ per switching, which is already competitive with state-of-the-art SOT-MRAM devices (Seen in Table S2). When the additional cost of generating an auxiliary field is taken into account, the advantage of our zero-field design becomes even more pronounced.

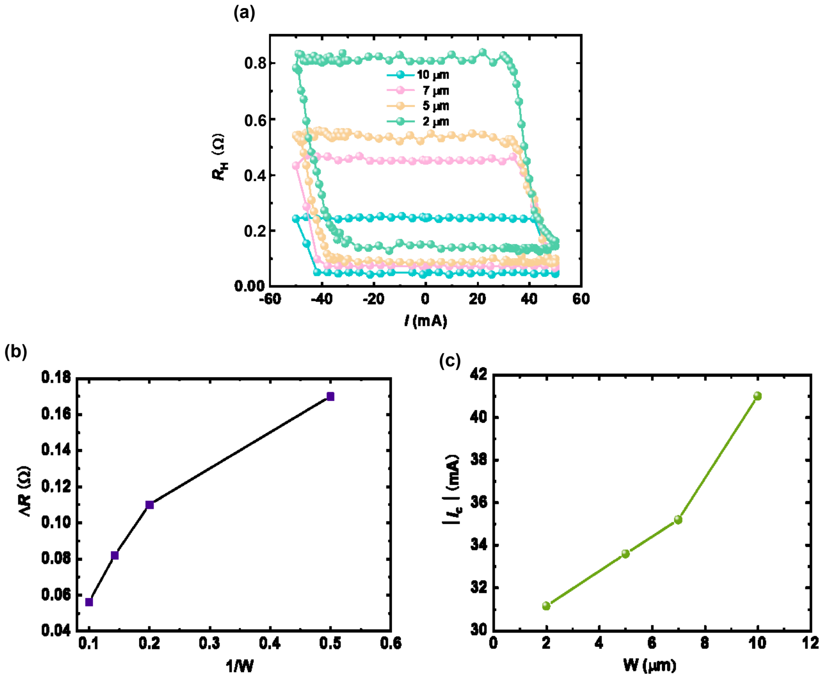

Figure S1. (a) SOT switching curves for different sizes (Width = 2, 5, 7, 10 μm). (b) the change of AHE resistances as a function of 1/W. (c) Absolute value of the threshold switching current as a function of W.

(3) **Comparison with literature:** We have added a comparison table (Table S2) summarizing the performance metrics of our device against state-of-the-art SOT-MRAM and neuromorphic devices reported in recent literature. The table highlights that while our proof-of-concept devices are relatively large, the scaling trends indicate that competitive performance can be achieved with further miniaturization.

Table S2: List of experimentally reported demonstrations of SOT-MTJ along with their key characteristics and properties

SOT materials	Free layer	Magnetic system	SOT width (nm)	Ic or Jc	Pulse width
Ta (5 nm)	CoFeB (1.4 nm)	In-plane	1200	10 MA/cm^2	500 ms
Ta (10 nm)	CoFeB (1.4 nm)	In-plane		8.2 MA/cm^2	40 ns to 100 ms
Pt (4 nm)	CoFeB (1.6 nm)	In-plane	1200	16 MA/cm^2	
Ta (6.2 nm)	CoFeB (1.6 nm)	In-plane	1000	2 mA	
W	CoFeB	In-plane	385	236 MA/cm^2	0.35 ns
Ta (20 nm)	CoFeB (1 nm)	PMA	1300	50 MA/cm^2	50 ns
Ta (3.8 nm)	CoFeB (1 nm)	PMA	220	0.5 mA	10 ms
W (3.5 nm)	CoFeB (1 nm)	PMA	170	130 MA/cm^2	0.3 to 10 ns
W (6 nm)	CoFeB (1 nm)	PMA	160	0.31 mA	0.3 to 10 ns
Pt (4 nm)	Co/CoFeB	PMA	170	0.4 mA	0.3 to 10 ns
Pt (3nm)	FeTb (6nm)	Field-free	2 μ-10 μm	40 mA	1 ms (This work)

Figure S1: Size-dependent properties of SAF;

Table S1: The calculated current distribution;

Table S2: List of experimentally reported demonstrations of SOT-MTJ along with their key characteristics and properties.
